# Multiple Myeloma Cell-Derived Secretome Modulates Bone Marrow Mesenchymal Stromal Cell Migration In Vitro

**DOI:** 10.3390/ijms27198763

**Published:** 2026-09-30

**Authors:** Maria João Fernandes, Andrea Giestinhas, Sara Reis Moura, Maria Helena Vasconcelos, Susana Gomes Santos, Maria José Oliveira, Maria Inês Almeida

**Affiliations:** 1 i3S, Instituto de Investigação e Inovação em Saúde, Universidade do Porto, Rua Alfredo Allen, 208, 4200-135 Porto, Portugal; 2 ICBAS, Instituto de Ciências Biomédicas Abel Salazar, Universidade do Porto, Rua de Jorge Viterbo Ferreira, 228, 4050-313 Porto, Portugal; 3 INEB, Instituto de Engenharia Biomédica, Universidade do Porto, Rua Alfredo Allen, 208, 4200-135 Porto, Portugal; 4 Laboratório de Microbiologia, Faculdade de Farmácia (FFUP), Universidade do Porto, Rua de Jorge Viterbo Ferreira, 228, 4050-313 Porto, Portugal; 5 UCIBIO, Laboratório de Bioquímica, i4HB, Faculdade de Farmácia (FFUP), Universidade do Porto, Rua de Jorge Viterbo Ferreira, 228, 4050-313 Porto, Portugal

**Keywords:** secretome, extracellular vesicles, cytokines/chemokines, bone marrow niche

## Abstract

Multiple myeloma (MM) depends on a permissive bone marrow microenvironment that supports malignant plasma cell survival and disease progression. Bone marrow mesenchymal stromal cells (BM-MSCs) are key components of this niche, but the mechanisms by which they are recruited by MM cells remain unclear. Here, we show that the MM-derived secretome (MM-Sec) promotes BM-MSC wound closure and transwell migration-associated responses in vitro. A small extracellular vesicle (sEV)-enriched pellet was isolated from MM.1S-Sec by differential ultracentrifugation and characterized, and the relative contributions of the sEV-enriched fraction and sEV-depleted secretome (post-100,000× *g*) to BM-MSC migration were assessed. The results show that the sEV-depleted secretome is more effective than the sEV-enriched fraction in promoting BM-MSC recruitment. Chemokine profiling of MM.1S-Sec identified CCL3 (MIP-1α) and CCL5 (RANTES) as abundant secreted factors and exploratory candidate contributors. Recombinant CCL3 and CCL5 increased the BM-MSC transwell migration response at high concentrations. Priming with MM.1S-Sec was associated with increased transwell migration-associated responses in the presence of CCL3 and CCL5. Together, these exploratory findings suggest that both vesicle-associated and soluble non-vesicular components may contribute to BM-MSC migration, while CCL3 and CCL5 may participate as candidate contributors within a broader network of soluble mediators. These findings identify secretome-mediated modulation of stromal migration as a relevant component of MM–microenvironment crosstalk that warrants further mechanistic investigation.

## 1. Introduction

Myeloma bone disease is among the most debilitating complications of multiple myeloma (MM), affecting approximately 85% of patients during the course of the disease [1]. It results from a profound imbalance between bone formation and resorption, leading to osteolytic lesions, pathological fractures and bone pain, the most common symptom at diagnosis [1]. Despite major therapeutic advances over the last two decades, MM remains an incurable disease, with most patients ultimately relapsing and developing drug resistance. The five-year relative survival is 60% [2], underscoring the urgent need for novel strategies that target both malignant cells and their supportive microenvironment.

The MM bone marrow microenvironment comprises a complex network of interacting cell types, including endothelial cells, immune cells, osteoclasts, osteoblasts and bone marrow mesenchymal stromal cells (BM-MSCs), which together create a supportive niche that promotes the survival and growth of malignant plasma cells [3,4]. Within this microenvironment, BM-MSCs play a central role in disease progression. However, their contribution remains controversial, as BM-MSCs can exhibit both tumor-supportive and tumor-suppressive functions depending on their activation state and context [5,6]. On one hand, BM-MSCs promote MM cell growth, survival and chemoresistance through direct cell–cell interactions and the secretion of pro-tumorigenic cytokines such as interleukin (IL)-6, Dickkopf-1 (DKK1), IL-1β, IL-3, granulocyte colony-stimulating factor (CSF), granulocyte–macrophage (GM)-CSF, stem cell factor and tumor necrosis factor (TNF)-α [5,6]. These soluble mediators promote adhesion, migration, and angiogenesis and disrupt bone remodeling, ultimately reinforcing tumor progression [7]. On the other hand, some studies suggest that BM-MSCs may exert anti-tumor effects by modulating immune responses or releasing inhibitory molecules, reflecting the complex and context-dependent role of these cells in MM [6].

Conversely, increasing evidence indicates that MM cells educate BM-MSCs, reprogramming them toward a pro-tumorigenic phenotype characterized by the secretion of factors that promote tumor survival, proliferation, migration and osteoclastogenesis, thereby contributing to the development of osteolytic bone lesions [5,8]. In line with this, MM patient-derived MSCs differ functionally from healthy or non-MM donor-derived MSCs [5], displaying reduced proliferative capacity, enlarged morphology and a premature senescence-associated phenotype [9,10,11], while secreting abnormally high levels of IL-6, IL-3, IL-1β, IL-10, TNF-α, OPN and DKK1 [9,12,13]. They also exhibit longer telomeres, increased IL-6 and macrophage inflammatory protein-1α (MIP-1α; CCL3) expression [14] and show impaired osteogenic differentiation due to sustained Notch signaling activation [15].

Given the critical role of BM-MSCs in shaping the MM niche and its progression, understanding their recruitment by MM cells is essential. In particular, investigating BM-MSC migration in response to a MM-derived secretome provides new insights into the role of MM-derived signals in modulating BM-MSC recruitment and may help identify communication pathways that could be targeted to disrupt tumor–stroma interactions in this still-incurable malignancy.

## 2. Results

### 2.1. Multiple Myeloma Secretome Promotes Mesenchymal Stromal Cell Wound Closure

**Figure 1:**
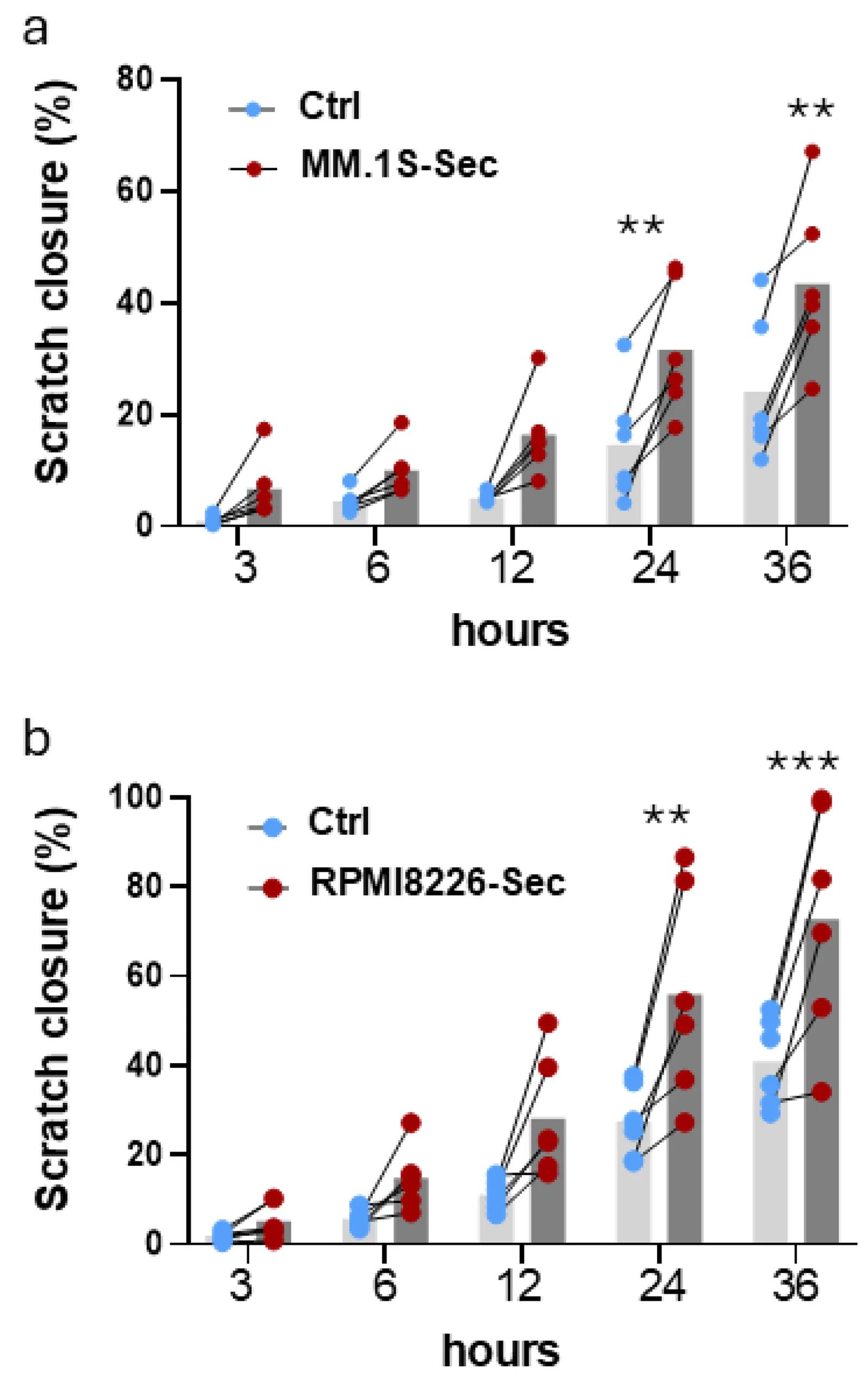
Scratch wound-healing assay showing bone marrow mesenchymal stromal cell (BM-MSC) migration in response to multiple myeloma secretome (MM-Sec). (**a**) BM-MSCs cultured with MM.1S-Sec and (**b**) RPMI8226-Sec, or controls (complete culture media). Light grey bars and blue dots represent control conditions (Ctrl), whereas dark grey bars and red dots represent MM-Sec conditions. Lines connect paired control and MM-Sec from the same experimental preparation. Data are presented as mean ± SEM of six independent experimental repeats (*n* = 6), performed using independently prepared multiple myeloma secretome batches and BM-MSC donor 1 at passage 5 (two-way ANOVA, Šídák’s multiple comparison test; ** *p* < 0.01, *** *p* < 0.001).

To determine whether the multiple myeloma secretome affects BM-MSC wound closure independently of direct cell–cell contact, a scratch wound-healing assay was performed. BM-MSC monolayers were scratched and cultured for up to 36 h in the presence of MM.1S or RPMI8226 secretome (MM.1S-Sec and RPMI8226-Sec, respectively). Cells cultured in RPMI supplemented with 10% (*v*/*v*) FBS served as the control. Exposure to the secretome from both MM cell lines increased BM-MSC wound closure relative to the corresponding control conditions from the early time points onward. However, statistically significant differences were detected only at 24 and 36 h. At these time points, MM.1S-Sec increased wound closure by 2.16-fold and 1.80-fold, respectively, relative to the corresponding time-matched controls (Figure 1a). Similarly, RPMI8226-Sec increased wound closure by 2.04-fold at 24 h and 1.78-fold at 36 h (Figure 1b). Proliferation was not directly assessed under each experimental condition; therefore, the relative contributions of migration and proliferation to wound closure cannot be distinguished. Overall, these results show that the secretome from both MM cell lines increase BM-MSC wound closure in vitro, with significant effects observed at the later time points.

### 2.2. Multiple Myeloma Secretome Increases the Mesenchymal Stromal Cell Transwell Migration Response

**Figure 2:**
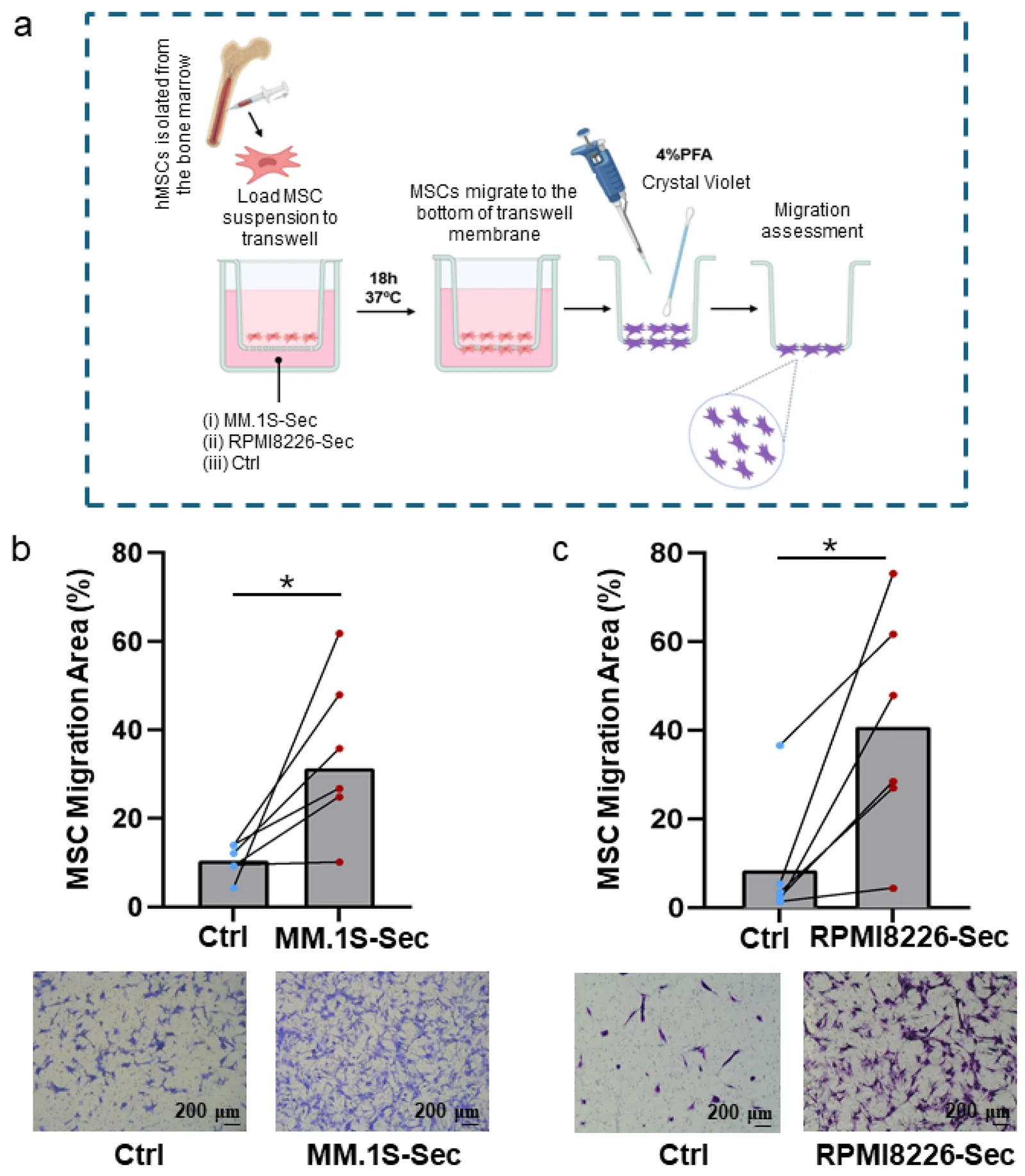
Transwell migration assay of bone marrow mesenchymal stromal cells (BM-MSCs) induced by multiple myeloma secretome (MM-Sec). (**a**) Schematic representation of the experimental design. (**b**) Migration induced by MM.1S-Sec, (**c**) migration induced by RPMI8226-Sec, and respective controls. Blue dots represent control conditions (Ctrl), whereas red dots represent MM-Sec conditions. Lines connect paired control and MM-Sec from the same experimental preparation. Data are presented as mean ± SEM of six independent experimental repeats (*n* = 6), performed using independently prepared multiple myeloma secretome batches and BM-MSC donor 1 at passages 5 and 6 (paired *t*-test; * *p* < 0.05). Objective ×5; scale bar, 200 micrometers.

To assess the BM-MSC transwell migration response to MM-derived secretome, BM-MSCs were seeded in the upper chamber of transwell inserts, and MM.1S-Sec or RPMI8226-Sec was placed in the lower compartment. Migration-associated staining was evaluated after 18 h (Figure 2a).

Both MM.1S-Sec and RPMI8226-Sec significantly promoted BM-MSC recruitment compared with control (RPMI + 10% (*v*/*v*) FBS). Specifically, MM.1S-Sec induced a 3.29-fold increase in the area covered by migrated BM-MSCs in the lower compartment (Figure 2b), whereas RPMI8226-Sec induced a 4.82-fold increase relative to the control (Figure 2c). Moreover, these findings confirm that MM-derived secretome enhances BM-MSC transwell migration, supporting the interpretation that the increased wound closure observed in the scratch assay reflects, at least in part, altered migratory behavior.

### 2.3. Multiple Myeloma-Derived Secretome Fractions Promote Mesenchymal Stromal Cell Migration

**Figure 3:**
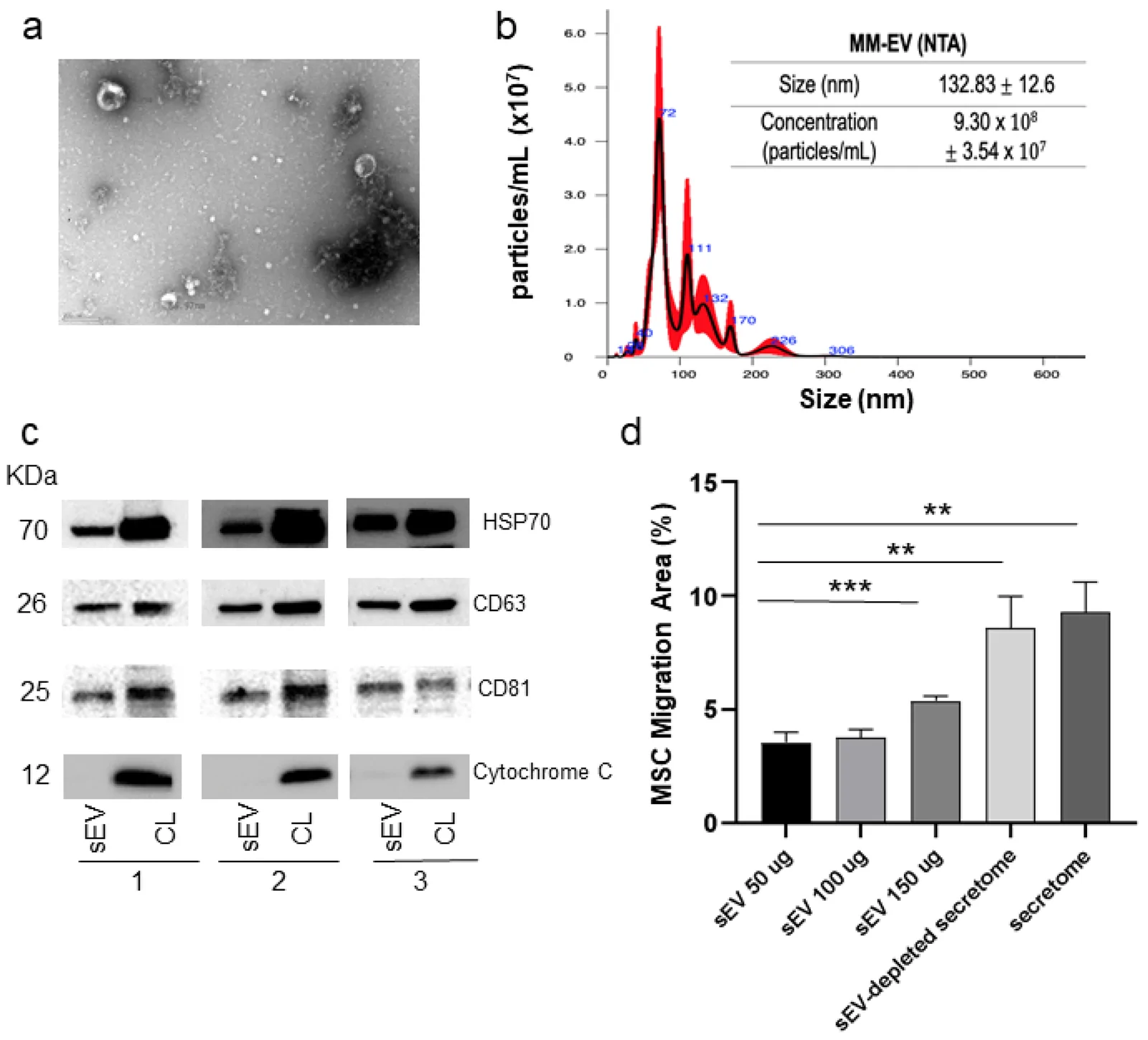
Characterization of small extracellular vesicles (sEVs) isolated from MM.1S and their effect on bone marrow mesenchymal stromal cell (BM-MSC) migration. (**a**) Transmission electron microscopy (TEM) image of sEVs (magnification: 50,000×; scale bar: 200 nanometers). Representative particle diameter is indicated. (**b**) Nanoparticle tracking analysis (NTA) showing sEV size (nm) and concentration (×10^7^ particles/mL) (*n* = 3). (**c**) Western blot of EV and cell lysates (CL) for the EV markers HSP70, CD63, and CD81, and the cellular marker cytochrome c (*n* = 3). (**d**) Effect of MM-derived sEV (50 µg, 100 µg, 150 µg), EV-depleted secretome (50 µg) and secretome (50 µg) and on BM-MSC migration. Data are presented as mean ± SEM of five independent experimental repeats (*n* = 5), performed using independently prepared multiple myeloma secretome batches and BM-MSC donors 2 and 3 at passage 5 (mean ± SEM; *n* = 5; one-way ANOVA, Dunnett’s multiple-comparison test, ** *p* < 0.01, *** *p* < 0.001).

Given the migratory responses observed following exposure to MM-Sec, we next investigated which secretome components might contribute to this activity. We isolated a small EV-enriched fraction (sEVs; 100,000× *g* pellet; 100k pellet) from MM.1S-Sec, which displayed a typical cup-shaped morphology when observed by transmission electron microscopy (TEM) (Figure 3a). The average diameter was 132.83 nm (Figure 3b), which falls within the expected size range of sEVs. To further characterize the EV-enriched preparation, specific EV-associated markers were assessed in accordance with the Minimal Information for Studies of Extracellular Vesicles guidelines [16]. The 100,000× *g* pellet was enriched in HSP70 and the tetraspanins CD63 and CD81 across three independent experiments. Cytochrome c, used as a marker of cellular contamination, was detected in cell lysates but not in the pellet preparation, supporting limited contamination by intracellular material (Figure 3c).

We next evaluated the effects of MM-derived sEVs and non-vesicular post-100,000× *g* fraction on BM-MSCs using a transwell recruitment assay (Figure 3d). MM-derived sEVs induced a dose-dependent increase in BM-MSC migration, with a 1.5-fold higher migratory response at 150 µg than at 50 µg. However, BM-MSC migration was significantly higher in the sEV-depleted secretome (or non-vesicular fraction of the secretome) at 50 µg, when compared to the corresponding sEV-containing fraction at 50 µg. Moreover, no significant difference in migration was observed between the non-vesicular fraction of the secretome and total secretome (Figure 3d); however, this result should not be interpreted as evidence of equivalence between these conditions. Moreover, we cannot exclude that the impact of 150 µg of sEV could be due to non-vesicular soluble mediators that may have co-precipitated with EV during ultracentrifugation. Together, these exploratory findings indicate that migration-associated activity is present in both the EV-enriched pellet and the post-100,000× *g* supernatant. They suggest a potential contribution from non-vesicular soluble factors but do not establish that these factors are the predominant drivers of BM-MSC migration or exclude a contribution from EV-associated components.

### 2.4. Profiling of Multiple Myeloma Secretome Identifies Candidate Chemokines Associated with Mesenchymal Stromal Cell Migration

**Figure 4:**
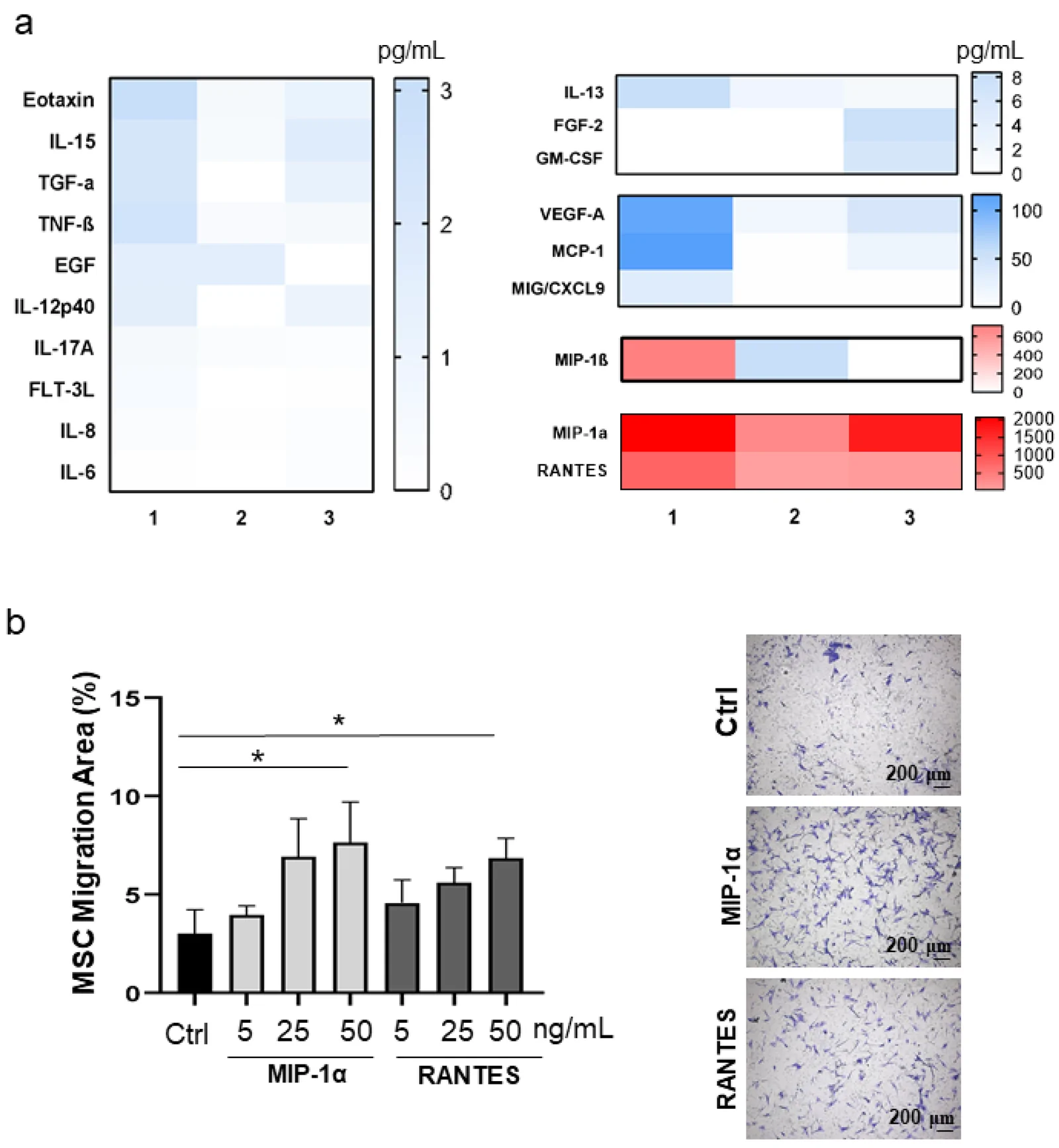
Chemokine/cytokine secretion by MM.1S cells. (**a**) Heatmap with levels of cytokines and chemokines secreted by MM.1S cells (*n* = 3 independent experiments; results in pg/mL). Panels use individual color-bar scales ranging 0–3 pg/mL up to 0–2000 pg/mL to accommodate differences in analyte abundance. (**b**) BM-MSC transwell migration-associated response in the presence of recombinant CCL3 or CCL5. Data are presented as mean ± SEM of five independent experimental repeats, using BM-MSC donor 3 at passage 5 (mean ± SEM; *n* = 5; Friedman test, Dunn’s multiple comparisons, * *p* < 0.05). Representative images showing BM-MSC migration towards control (Ctrl), 50 ng/mL of CCL3, and 50 ng/mL CCL5 (top to bottom, respectively). Objective ×5; scale bar, 200 micrometers.

To characterize the cytokines and chemokines present in MM.1S-conditioned medium, we analyzed a panel of 48 analytes. Unconditioned RPMI containing 10% (*v*/*v*) FBS, incubated for 48 h under the same conditions but without cells, was analyzed as a matched medium-background control. The control-medium concentration for each analyte was subtracted from the corresponding MM.1S-conditioned-medium concentration. Values below zero after subtraction were assigned a value of 0 pg/mL. This comparison estimates the increase in analyte concentration following conditioning but does not distinguish active secretion from contributions associated with cell death or other culture-related changes. Across three independent experiments, CCL3 (MIP-1α) and CCL5 (RANTES) emerged as the most abundantly secreted chemokines, with mean concentrations of 1361.18 pg/mL and 299.85 pg/mL, respectively (Figure 4a). VEGF and MCP-1 (CCL2) were detected at intermediate levels, with mean concentrations of 55.33 pg/mL and 45.77 pg/mL, respectively. Several additional chemokines/cytokines, including IL-13, IL-15, IL-17A, TNF-β, MIG/CXCL9 and Eotaxin, were consistently increased across the three experiments, although at lower concentrations (<10 pg/mL) (Figure 4a). Chemokines/cytokines that were not detected or were below the limit of detection are listed in Supplemental Table S1.

Next, we examined whether CCL3 and CCL5, the two most abundant secreted chemokines in MM.1S-Sec, could increase BM-MSC transwell migration response. Both factors dose-dependently increased BM-MSC migration, with significant increases at 50 ng/mL, increasing 2.52-fold (CCL3) and 2.25-fold (CCL5), compared with the negative control (Figure 4b). These findings indicate that CCL3 and CCL5 can promote BM-MSC transwell migration response but only at concentrations substantially higher than those detected in the MM secretome. This suggests that these chemokines alone are insufficient to account for the full migratory response and that additional components of the MM secretome may be required to potentiate their effects on BM-MSC migration.

**Figure 5:**
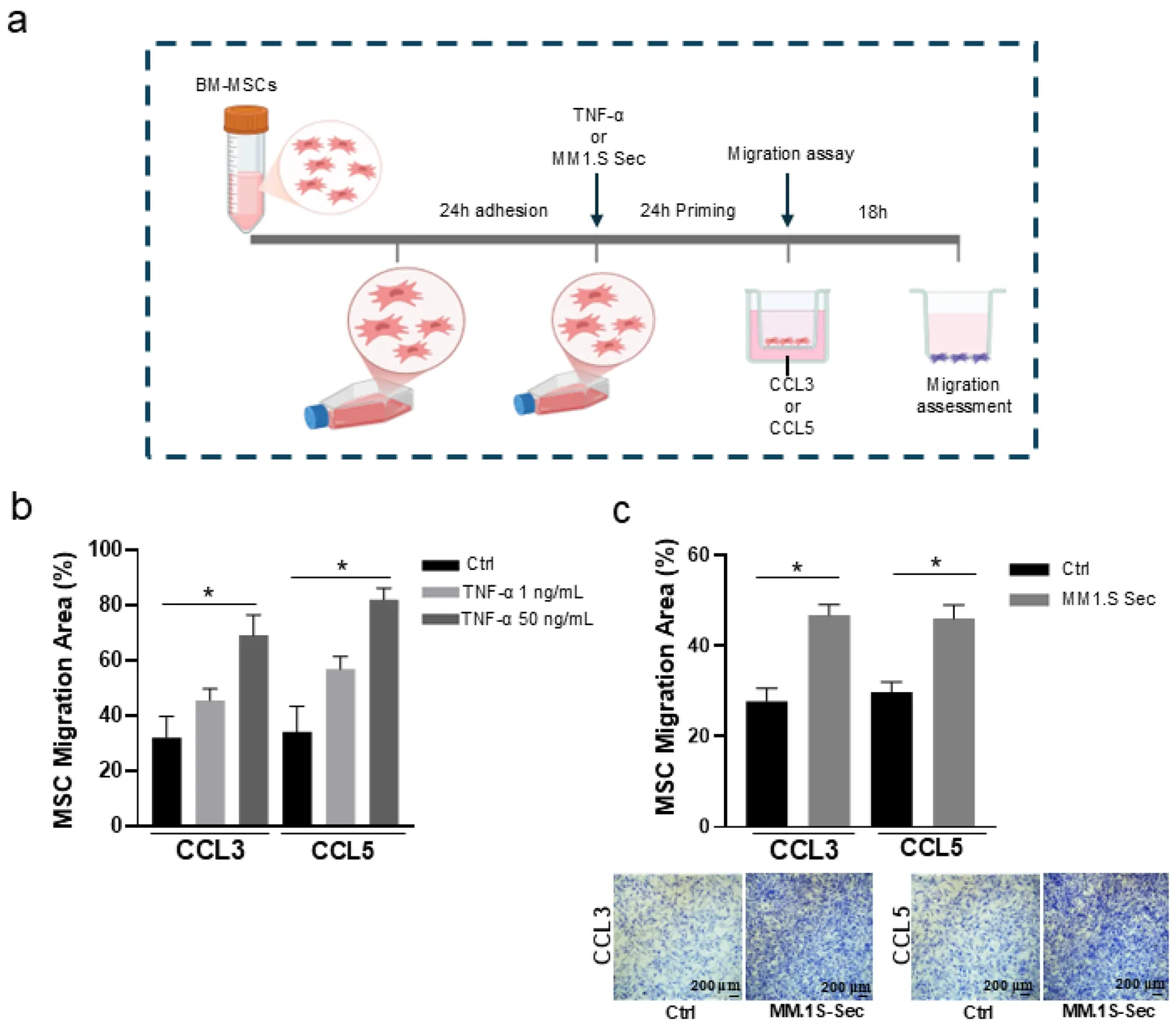
MM.1S secretome and TNF-α priming effects in bone marrow mesenchymal stromal cell (BM-MSC) migration toward CCL3 and CCL5. (**a**) Experimental set-up. (**b**) BM-MSCs were primed with RPMI alone or with TNF-α (1 or 50 ng/mL), and the transwell migration-associated response was subsequently assessed with CCL3 (50 ng/mL) or CCL5 (50 ng/mL) in the lower chamber. Data are presented as mean ± SEM of four independent experimental repeats, using BM-MSC donor 3 at passage 6 (mean ± SEM; *n* = 4; Friedman test, Dunn’s multiple comparisons, * *p* < 0.05). (**c**) BM-MSCs were primed with RPMI supplemented with 10% (*v*/*v*) FBS or with MM.1S secretome (MM.1S-Sec), followed by assessment of the transwell migration-associated response with CCL3 or CCL5 in the lower chamber. Data are presented as mean ± SEM of four independent experimental repeats, using BM-MSC donor 3 at passage 6 (mean ± SEM; *n* = 4; Friedman test, Dunn’s multiple comparisons; * *p* < 0.05). Representative images of migration assays of BM-MSCs induced by CCL3 and CCL5 after priming. Objective ×5; scale bar, 200 micrometers.

As BM-MSCs within the MM bone marrow microenvironment are continuously exposed to tumor-derived signals that may alter their functional state, we next performed a priming assay to determine whether prior MM-associated activation enhances BM-MSC responsiveness to these chemokines (Figure 5a). BM-MSCs were preconditioned with TNFα as a positive control, as TNFα preincubation has been reported to enhance MSC migration toward chemokines [17]. TNF-α priming enhanced BM-MSC migration toward both CCL3 and CCL5, with the strongest effect observed at 50 ng/mL TNF-α, which resulted in statistically significant 2.40-fold and 2.85-fold increases, respectively, relative to non-primed cells (cultured with medium alone for 24 h) (Figure 5b). Priming with a lower concentration of TNF-α (1 ng/mL) also increased BM-MSC migration by 1.71-fold toward CCL3 and 2.18-fold toward CCL5, although these effects did not reach statistical significance (Figure 5b). Together, these findings indicate that inflammatory priming enhances BM-MSC responsiveness to both chemokines. Next, we investigated whether priming with the cocktail of chemokines/cytokines present in MM secretome could modulate BM-MSC migration. Priming with MM.1S-Sec for 24 h significantly enhanced chemokine-driven migration compared with non-primed control cells, resulting in 3.11-fold and 3.06-fold increases toward CCL3 and CCL5, respectively (Figure 5c). These results show that MM.1S-derived soluble factors potentiate BM-MSC transwell migration response to CCL3 and CCL5. Collectively, our data indicate that MM.1S-Sec primes BM-MSCs for an enhanced migration response to these chemokines.

## 3. Discussion

In this study, we show that MM-derived secretome promote BM-MSC migration and that this effect is primarily mediated by soluble components, while MM-derived EVs (at 150 µg) also independently modulate BM-MSC migratory behavior. These findings support the view that the MM secretome is a key regulator of stromal cell recruitment within the bone marrow microenvironment. In addition, we identified CCL3 (MIP-1α) and CCL5 (RANTES) as abundant MM-derived chemokines and candidate mediators of BM-MSC migration, and found that prior exposure to MM-secretome enhances BM-MSC responsiveness to these signals. Together, these data suggest that MM cells not only attract BM-MSCs through secreted factors, but also alter their migratory responsiveness, thereby potentially facilitating stromal recruitment and microenvironmental remodeling.

The MM secretome, comprising soluble factors, EV-associated cargo, and other secreted components, represents a complex signaling network that shapes the bone marrow microenvironment [7,18,19]. Although EVs contain a broad repertoire of bioactive proteins, lipids, and nucleic acids capable of modulating recipient cells [18], our results show that migration-associated activity was present in both the small-EV-enriched 100,000× *g* pellet and the post-100,000× *g* supernatant. These findings suggest that vesicle-associated and non-vesicular components may both contribute to the BM-MSC transwell migration response. However, their relative contributions, potential cooperation and effects on stromal cell trafficking were not established in the present study. In other cellular contexts, MM-derived EVs have been shown to be internalized by monocytes, promoting their differentiation into pro-tumoral macrophages that subsequently enhance MM cell proliferation, migration, and survival, underscoring their broader contribution to the establishment of a tumor-supportive niche [20,21]. Among the soluble mediators identified in our study, CCL3 and CCL5 emerged as two of the most abundantly secreted chemokines by MM.1S cells. Both are well known to participate in cell recruitment and microenvironment remodeling [22,23], making them strong candidates for functional validation. However, although recombinant CCL3 and CCL5 significantly promoted BM-MSC migration, this effect was observed only at concentrations substantially higher than those measured in MM.1S-secretome. Therefore, the levels of these chemokines present in the MM.1S secretome are unlikely to be sufficient, on their own, to account for the full migratory response. Rather, CCL3 and CCL5 may contribute to BM-MSC recruitment in cooperation with other soluble mediators present in the MM secretome.

A limitation of the present study is that the functional requirement for CCL3 and CCL5 in MM-secretome-induced BM-MSC recruitment was not directly tested. Although these chemokines were among the most abundant factors detected in the MM.1S secretome and recombinant CCL3 and CCL5 promoted BM-MSC migration, definitive validation of their mechanistic contribution will require assessment of the corresponding receptors, including CCR1 and CCR5, in BM-MSCs, as well as neutralization, receptor inhibition, or chemokine-depletion experiments using MM-conditioned media. Another limitation of this study is that chemokine/cytokine profiling was performed only on MM.1S-conditioned medium, whereas the pro-migratory effect of MM-derived secretome was observed using both MM.1S and RPMI8226 cells. Therefore, the secretome profiling results, including the identification of CCL3 and CCL5 as abundant chemokines, should be interpreted as specific to MM.1S under the experimental conditions used. Future profiling of RPMI8226-conditioned medium will be required to determine whether these candidate mediators are shared across MM cell lines or reflect a cell line-specific secretory profile. In addition, our multiplex assay was limited to 48 analytes, and we therefore cannot exclude the involvement of other relevant mediators not represented in this panel. For example, TECK/CCL25 has been reported to be highly secreted by RPMI8226 cells and to promote MSC migration [24], while the same study also identified elevated CCL5/RANTES secretion, consistent with our findings. An additional limitation concerns the production and normalization of the conditioned medium. The unconditioned control medium was incubated for the same duration and under the same environmental conditions as the conditioned medium but did not undergo changes associated with cell growth and metabolism. End-point viable cell number, cell death, and pH were not systematically assessed, and analyte concentrations were not normalized to the number of viable producer cells. Therefore, nutrient depletion, metabolite accumulation, pH changes, and material released by non-viable cells may have contributed to the biological effects of the conditioned medium. Consequently, the observed activity cannot be attributed exclusively to actively secreted signaling factors.

An important finding of this study is that MM-conditioned medium not only acts as a chemoattractant, but also primes BM-MSCs for enhanced migration in response to chemokine stimulation. BM-MSCs exposed to MM.1S-conditioned medium exhibited an exaggerated migratory response to both CCL3 and CCL5, indicating that soluble MM-derived factors can rapidly alter stromal cell behavior even in the absence of direct cell–cell contact. This is particularly relevant in the context of the MM bone marrow niche, where stromal cells are continuously exposed to tumor-derived signals [25]. Inflammatory mediators are likely to contribute to this process, as TNF-α priming enhanced BM-MSC responsiveness to both chemokines. In this study, TNF-α was used as a positive control to test the concept that inflammatory signals [17] can prime BM-MSCs to acquire a migratory phenotype. Notably, TNF-α was not detected in MM.1S secretome under our experimental conditions, or was present only at levels below the detection limit. Therefore, our findings do not suggest that MM.1S cells are a direct source of TNF-α. Instead, TNF-α is known to be elevated in multiple myeloma at the level of the bone marrow microenvironment, where it is generated by other cellular components of the niche, and contributes to stromal activation and microenvironmental remodeling [26,27]. Our findings therefore support a model in which inflammatory signals present in the MM microenvironment participate in the early education of BM-MSCs, increasing their responsiveness to chemotactic cues and favoring tumor-supportive interactions.

This concept is consistent with previous reports showing that BM-MSCs derived from patients with MM are transcriptionally and functionally distinct from those of healthy donors [28,29]. Fernando et al. demonstrated that MM-derived BM-MSCs retain persistent gene expression alterations even after in vitro expansion, suggesting that MM-associated changes are stably imprinted and maintained independently of the immediate tumor microenvironment [29]. Likewise, Alameda et al. reported that freshly isolated BM-MSCs from MM patients display more than 600 differentially expressed genes compared with healthy donor BM-MSCs, further supporting the existence of a disease-associated stromal phenotype [28]. Functionally, MM-derived BM-MSCs exhibit reduced proliferative capacity, altered secretion of growth factors, cytokines, and chemokines, and features of premature senescence [30]. In the present study, the use of non-MM donor BM-MSCs was a deliberate experimental choice, as our aim was to model early stromal recruitment events and to determine whether MM-derived soluble factors are sufficient to modulate the migratory behavior of BM-MSCs that have not been chronically exposed to, or previously educated by, the MM bone marrow microenvironment. This approach allowed us to assess the direct effect of MM-derived secretome on BM-MSC migration without the confounding influence of disease-associated stromal alterations. Nevertheless, we acknowledge that BM-MSCs derived from MM patients may display disease-associated phenotypic and functional changes that could alter their migratory response. Future studies using MM patient-derived BM-MSCs will therefore be required to determine whether the responses observed here are maintained, enhanced, or altered in disease-educated stromal cells. It is therefore plausible that BM-MSCs chronically exposed to the MM microenvironment in patients acquire more deeply imprinted, and potentially non-reversible, alterations in their migratory and inflammatory programs [28,31]. Such changes may reinforce tumor–microenvironment crosstalk and contribute to disease progression.

Finally, our findings should be interpreted in the context of existing evidence showing that MM cells and BM-MSCs also establish direct physical interactions. In co-culture systems, MM cells adhere to BM-MSCs, providing clear evidence of direct cell–cell communication [32]. Our study demonstrates that paracrine signaling is a major component of this interaction network. Still, future work should integrate both contact-dependent and soluble factor-mediated mechanisms in more physiologically relevant models, including advanced three-dimensional bone marrow niche systems that more faithfully recapitulate in vivo conditions. Such models will be essential to better understand how chemotactic cues, stromal priming, and direct tumor–stroma interactions cooperate during MM progression.

## 4. Materials and Methods

### 4.1. Cell Lines

Human malignant B lymphocyte MM.1S cells (ATCC, CRL-2974) and RPMI8226 cells (ATCC, CCL-155, Manassas, VA, USA) were cultured in Roswell Park Memorial Institute (RPMI) 1640 Medium (Corning, NY, USA)) with 10% (*v*/*v*) heat-inactivated fetal bovine serum (FBS) (Biowest, Nuaillé, France) at 37 °C and 5% (*v*/*v*) carbon dioxide (CO_2_). Cell lines were routinely tested for mycoplasma contamination every three months, and all experiments were performed using mycoplasma-negative cultures. For experiments with MM.1S and RPMI8226 secretome (MM-Sec), MM.1S and RPMI8226 cells were cultured at a density of 2 × 10^5^ cells/mL for 48 h. The secretome was then collected, followed by centrifugation at 300 *g* to remove cells from the supernatant and 2000× *g* to discard cell debris. Supernatants were stored at −80 °C until further use. Complete culture media (RPMI + 10% (*v*/*v*) FBS), without any contact with cells, were incubated in the same conditions and used as a control. Specifically, the unconditioned control medium was incubated for 48 h under the same temperature and CO_2_ conditions as the cell-conditioned medium. However, unlike the conditioned medium, the control medium was not exposed to changes associated with cell growth, including nutrient consumption, metabolite accumulation, alterations in pH, or the release of material from non-viable cells. For the purposes of this study, an “independent experiment” was defined as an assay performed on a separate day using a distinct culture of multiple myeloma cells and an independently prepared batch of multiple myeloma cell-conditioned medium. Different cell passages were used where applicable.

### 4.2. Human Primary Bone Marrow-Derived Mesenchymal Stromal Cells

Primary human BM-MSC were isolated from the BM of patients who had undergone surgery for an anterior cruciate ligament injury and patients who had undergone surgery for fragility fractures (donor 1: male; age < 37 years; site of bone marrow collection: anterior tibia) or surgery for fragility fractures of the hip (donor 2: female; age 75 years; site of bone marrow collection: hip; donor 3: male; age 70 years; site of bone marrow collection: hip). None of the patients had any known inflammatory diseases. Characterization of BM-MSCs was performed as previously described [33,34,35] and in accordance with the criteria established by the International Society for Cellular Therapy [36]. The experiments were performed between passages 5 and 9. The cells were maintained in Dulbecco’s Modified Eagle’s Medium (DMEM) with low glucose, supplemented with 10% (*v*/*v*) FBS (MSC qualified, Gibco, Grand Island, NY, USA).

Throughout the manuscript, MM-derived secretome (MM-Sec) refers to the unfractionated cell-free conditioned medium collected from MM cells after 48 h of culture. MM.1S-Sec and RPMI8226-Sec refer to secretome derived from the MM.1S and RPMI8226 cell lines, respectively. Control medium refers to RPMI supplemented with 10% (*v*/*v*) FBS incubated under the same conditions in the absence of cells.

### 4.3. Scratch Wound Assay

BM-MSC were seeded in 48-well plates at a cell density of 0.4 × 10^5^ cells/mL and left to adhere for 24 h. Subsequently, a straight scratch was carefully created using a 20 µL pipette tip, simulating a wound. The supernatant was aspirated, and any detached cells present were subsequently removed. BM-MSCs were then incubated with CM derived from MM.1S or RPMI8226 cells, or control medium (RPMI + 10% [*v*/*v*] FBS), and microscope images were captured at 3, 6, 12, 24 and 36 h, allowing for the quantification of scratch closure. The remaining open area resulting from the wound was measured using x [37].

### 4.4. Transwell Migration Assay

Transwell inserts with 8 μm transparent PET membranes (BD Falcon, Franklin Lakes, NJ, USA) were hydrated with 500 µL of DMEM for 30 min at 37 °C in 24-well plates before cell seeding. Subsequently, 60,000 BM-MSCs were resuspended in 500 μL of DMEM serum-free media and placed in each well on top of the membranes (top compartment, TC). The bottom compartment (BC) contained 750 µL of distinct stimuli, namely (i) MM-derived secretome from MM.1S or RPMI8226, (ii) complete culture media (RPMI + 10% [*v*/*v*] FBS), (iii) MM.1S-derived EVs (50 µg, 100 µg, 150 µg), (iv) EV-depleted secretome from MM.1S (50 µg), (v) secretome from MM.1S (50 µg), (vi) 5 ng/mL, 25 ng/mL, or 50 ng/mL CCL3 (Gibco, 300-08-20UG), or (vii) 5 ng/mL, 25 ng/mL or 50 ng/mL CCL5 (Gibco, 300-06-20UG). After 18 h, cells were fixed with 4% (*w*/*v*) paraformaldehyde and stained with 0.05% (*w*/*v*) crystal violet. Cells remaining on the upper membrane surface were removed using cotton swabs. For each Transwell insert, six randomly selected, non-overlapping microscopic fields from the lower membrane surface were imaged using a Zeiss Axiovert 2000M microscope (Zeiss, Oberkochen, Germany) with a 5× objective. Identical microscope settings and magnification were used for all experimental groups. Migration-associated staining was quantified as the crystal-violet-positive area using ImageJ Version 1.54p. The quantified end-point therefore represents migration-associated stained area rather than a direct count of migrated cells. Image acquisition and analysis were performed blinded to the experimental condition.

### 4.5. Isolation and Characterization of Extracellular Vesicles

For EV collection, 30 × 10^6^ MM.1S cells were cultured at a density of 2 × 10^5^ cells/mL in RPMI 1640 supplemented with 2% (*v*/*v*) EV-depleted FBS, for 48 h. The EV-depleted FBS was prepared by ultracentrifugation to reduce bovine-derived EV contamination. The isolation of EV was performed through a series of differential centrifugation steps of the secretome, as follows: the supernatant was centrifuged at 300× *g* for 5 min at RT to remove cells; the resulting supernatant was sequentially centrifuged at 2000× *g* for 10 min at 4 °C, collected, and centrifuged again at 10,000× *g* for 20 min at 4 °C to remove cell debris and the 10K EV pellet; the supernatant was centrifuged at 100,000× *g* for 2 h at 4 °C in an ultracentrifuge (Beckman Optima XE-100, Beckman, Brea, CA, USA; 70 Ti fixed angle rotor; Beckman polycarbonate bottle with cap assembly) to collect the 100K EV pellet [38]. Lastly, the EV pellet was resuspended in particle-free 1× PBS, and stored at −80 °C in aliquots to avoid repeated freeze–thaw cycles. The supernatant was collected as an experimental control representing the EV-depleted secretome. The protein content of the samples was quantified using the DC Protein Assay Kit (Bio-Rad, Hercules, CA, USA) and used for the transwell assays. The total secretome corresponds to the pre-100,000× *g* aliquot from the preparation containing 2% (*v*/*v*) FBS. The sEV-enriched fraction corresponds to the 100,000× *g* pellet, whereas the sEV-depleted secretome corresponds to the post-100,000× *g* supernatant from the same 2% FBS preparation. Transwell assays were conducted using MM.1S-derived EVs (100,000× *g* pellet; 50 µg, 100 µg, 150 µg) or EV-depleted secretome from MM.1S (100,000× *g* supernatant; 50 µg) or secretome from MM.1S (50 µg).

Transmission electron microscopy (TEM) was used to visualize the morphology of EVs using a Jeol JEM 1400 electron microscope (JEOL Ltd., Tokyo, Japan). The samples were loaded onto 400-mesh formvar nickel grids for 1 min, followed by negative staining with 5% (*w*/*v*) uranyl acetate for 30 s. Nanoparticle Tracking Analysis (NTA) was used to assess the size and concentration of EVs using a NanoSight NS300 and NTA 3.2 software (Malvern Panalytical, Worcestershire, UK). Successful EV depletion of FBS was confirmed by both TEM and NTA.

### 4.6. Western Blot

MM.1S cells lysates were obtained by incubating cells with RIPA buffer (20 mM Tris-HCl pH 7.5, 150 mM NaCl, 1% (*v*/*v*) Triton X-100 and 1% (*v*/*v*) Nonidet P-40 (Sigma-Aldrich, St. Louis, MO, USA)) in addition with protease inhibitors (10 μL/mL Phenylmethylsulfonyl fluoride (PMSF, 200 mM), 10 μL/mL Aprotinin (1 mg/mL) and 10 μL/mL Leupeptin (1 ng/mL)) and phosphatase inhibitors (20 μL/mL NaVO_3_ (50 nM), 50 μL/mL Na_3_P_2_O_7_ (50 mg/mL) and 10 μL/mL NaF (10 mM)), for 30 min, gently mixing by inversion every 10 min. The samples were then centrifuged at 20,000× *g* for 10 min at 4 °C and the supernatant collected. EV pellets and cell lysates were denatured at 95 °C for 5 min and resolved in 10% (*w*/*v*) SDS-PAGE polyacrylamide gels, in reducing conditions (with β-mercaptoethanol). Thereafter, the proteins were transferred to nitrocellulose membranes, blocked with 5% (*w*/*v*) milk in TBS-T 0.1% (*v*/*v*), and incubated overnight with the primary antibodies for CD63 (1:1000, EXOAB-CD63A-1), CD81 (1:1000, EXOAB-CD81A-1), HSP70 (1:1000, EXOAB-Hsp70A-1) (System Biosciences, Palo Alto, CA, USA) and Cytochrome C (1:1000, sc-13156, Santa Cruz Biotechnology, Dallas, TX, USA). On the next day, the membranes were incubated with HRP-conjugated secondary antibodies (1:2000, anti-rabbit, EXOAB-CD63A-1 System Biosciences, and 1:2000, anti-mouse, NA931-1ML Cytiva, Marlborough, MA, USA) for 1 h. Membranes were incubated with ECL substrates (Bio-Rad) and the chemiluminescent signal was detected through ChemiDoc Imaging System (Bio-Rad) (Supplemental Figure S1).

### 4.7. Quantification of Chemokines and Cytokines

MM.1S secretome from MM.1S (250 μL), collected from three independent experiments, and the respective control media (RPMI supplemented with 10% ([*v*/*v*] FBS) were analyzed for their chemokine and cytokine profiles using the Human Cytokine/Chemokine 48-Plex Discovery Assay (Eve Technologies Corp., Calgarym, AB, Canada). For each preparation and analyte, the concentration measured in the matched unconditioned control medium was subtracted from that measured in MM.1S-conditioned medium. Background-subtracted values below zero were assigned a value of 0 pg/mL. Analyte-specific limits of detection and lower limits of quantification were obtained from the assay provider. Values reported by the assay provider as below the limit of detection were not assigned a numerical concentration and were reported as <LOD. Cytokines/chemokines below the assay detection or quantification limits are described in Supplemental Table S1.

### 4.8. Priming Assay

BM-MSCs were seeded in DMEM with low glucose, supplemented with 10% (*v*/*v*) FBS (MSC qualified, Gibco) at 0.24 × 10^6^ cells per condition and incubated overnight in DMEM with low glucose, supplemented with 10% (*v*/*v*) FBS (MSC qualified, Gibco) to allow adherence. The following day, culture medium was replaced by (i) RPMI without serum (Ctrl), (ii) RPMI without serum supplemented with TNF-α at 1 ng/mL, (iii) RPMI without serum supplemented with TNF-α at 50 ng/mL, (iv) complete medium RPMI with 10% (*v*/*v*) FBS (Ctrl), or (v) MM.1S-secretome (MM1S-Sec), and BM-MSCs were incubated for 24 h. Following conditioning, BM-MSCs were washed, detached and resuspended in RPMI serum-free media for transwell experiments. For each condition, 0.06 × 10^6^ MSCs were plated per transwell in the upper compartment.

### 4.9. Statistical Analysis

Statistical analyses were performed using GraphPad Prism version 9.0.0 (GraphPad Software, Inc.). Data are presented as the mean ± standard error of the mean (SEM), and n denotes the number of independent experimental repeats. Data distribution was assessed using the Shapiro–Wilk normality test. Scratch wound-closure data were analyzed using two-way repeated-measure ANOVA, followed by Šídák’s multiple-comparison test to compare control and secretome-treated conditions at each time point. Comparisons between two paired groups were performed using a paired Student t-test. For comparisons among more than two matched groups that did not meet the assumptions of parametric testing, the Friedman test followed by Dunn’s multiple-comparison test was used. One-way ANOVA followed by the appropriate multiple-comparison test was used for comparisons among more than two groups when the data met the assumptions of parametric testing. Statistical significance was defined as *p* < 0.05. Significance levels are indicated as follows: * *p* < 0.05, ** *p* < 0.01, and *** *p* < 0.001.

## 5. Conclusions

In conclusion, our results show that MM-derived secretome increase BM-MSC migration-associated responses in vitro, with CCL3 and CCL5 emerging as candidate contributors that may participate in, but do not fully account for, this effect. Although MM-derived sEVs exert complementary actions, the overall BM-MSC migration response is driven by a broader combination of secreted molecules. A better understanding of these early interactions between MM cells and BM-MSCs may help identify future strategies to interfere with tumor–stroma crosstalk in MM, although direct inhibition studies will be required to evaluate therapeutic relevance.

## Data Availability

The data that support the findings of this study are available from the corresponding author upon reasonable request.

## Supplementary Materials

The following supporting information can be downloaded at: https://www.mdpi.com/article/10.3390/ijms27198763/s1.
