# Supplementary material for: Multiple Myeloma Cell-Derived Secretome Modulates Bone Marrow Mesenchymal Stromal Cell Migration In Vitro
Source: Int J Mol Sci. 2026 Sep 30;27(19):8763. doi: 10.3390/ijms27198763 (PMC13665306; doi:10.3390/ijms27198763)
Supplement: Supplementary file 1 [file ijms-27-08763-s001.zip › ijms-4363925-supplementary.pdf]

Supplemental Table S1. List of cytokines/chemokines that were not detected or below detected limit.

Non-detected or below detected limit cytokines
IL-1α
IL-1β
IL-1RA
IL-2
GROα
IFN-α2
IFNγ
Fractalkine
G-CSF
GM-CSF
IL-5
IL-4
IL-3
IL-7
IL-9
IL-10
IL-12p70
IL-17E/IL-25
IL-17F
IL-18
IL-22
IL-27
IP-10
MCP-3
M-CSF
MDC
PDGF-AA
PDGF-AB/BB
TNFα

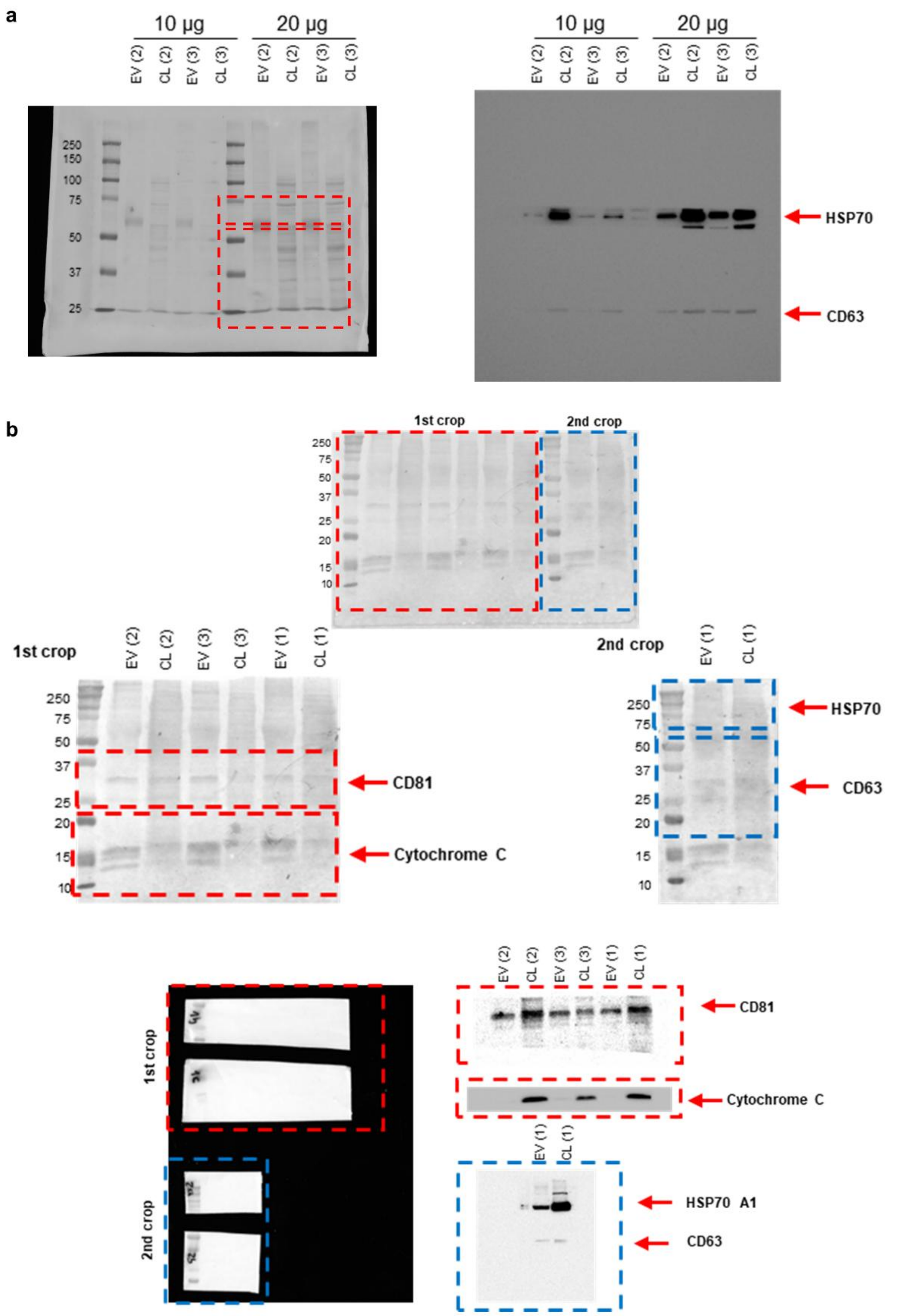

Supplemental Figure S1. Full-length membranes and cropped regions used for western blot characterization of the EV-enriched pellet.
